# Supplementary material for: End Sequence Analysis Toolkit (ESAT) expands the extractable information from single-cell RNA-seq data
Source: Genome Res. 2016 Oct;26(10):1397–410. doi: 10.1101/gr.207902.116 (PMC5052061; doi:10.1101/gr.207902.116)
Supplement: Supplemental Material [file supp_gr.207902.116_Supp_Materials.tar.gz › ESAT-esat_v0.1/doc/html/classbroad_1_1core_1_1multiplealignment_1_1_m_a_f_alignment.html]

ESAT: broad.core.multiplealignment.MAFAlignment Class Reference


ESAT


- Main Page
- Related Pages
- Packages
- Classes
- Files

- Class List
- Class Index
- Class Hierarchy
- Class Members

All Classes Namespaces Files Functions Variables Enumerator Pages

- broad
- core
- multiplealignment
- MAFAlignment

Classes |
Public Member Functions |
Static Public Member Functions |
Static Public Attributes |
Protected Member Functions |
List of all members

broad.core.multiplealignment.MAFAlignment Class Reference

Inheritance diagram for broad.core.multiplealignment.MAFAlignment:

[legend]

Collaboration diagram for broad.core.multiplealignment.MAFAlignment:

[legend]

|  |  |
| --- | --- |
| Classes | |
| class | **MAFHeader** |
|  | |
| class | **MAFMultipleAlignmentBlock** |
|  | |

|  |  |
| --- | --- |
| Public Member Functions | |
|  | MAFAlignment () |
|  | |
|  | MAFAlignment (String indexFile) throws IOException, ParseException |
|  | |
| MAFHeader | getHeader () |
|  | |
| void | encode () |
|  | |
| void | encodeAsMatrix () |
|  | |
| void | reverse () |
|  | |
| int | length () |
|  | |
| Map< String, Short > | getColumn (int i) |
|  | |
| Map< String, Matrix > | getColumnsAsVector (int start, int number) |
|  | |
| void | addShortEncodedColumn (Map< String, Short > col) |
|  | |
| void | addShortEncodedRegion (Map< String, short[]> region) |
|  | |
| void | addSequence (AlignedSequence seq) |
|  | |
| void | addSequences (List< AlignedSequence > sequences) |
|  | |
| boolean | isEmpty () |
|  | |
| void | write (BufferedWriter bw) throws IOException |
|  | |
| MAFAlignment | getSubAlignment (int refStart, int refEnd, boolean reverse) |
|  | |
| AlignedSequence | getAlignedSequence (String sequenceId, boolean fillInGapsBetweenBlocks) |
|  | |
| AlignedSequence | getAlignedSequence (String sequenceId) |
|  | |
| List< AlignedSequence > | getAlignedSequences (boolean fillGapsBetweenBlocks) |
|  | |
| List< AlignedSequence > | getAlignedSequences () |
|  | |
| boolean | overlaps (LightweightGenomicAnnotation annotation) |
|  | |
| boolean | contains (LightweightGenomicAnnotation annotation) |
|  | |
| List< String > | getAlignedSequenceIds () |
|  | |
| int | getReferenceStart () |
|  | |
| int | getReferenceEnd () |
|  | |
| void | compress () |
|  | |
| MultipleAlignment | toMultipleAlignment (boolean fillGapsBetweenBlocks) |
|  | |
| MultipleAlignment | toMultipleAlignment () |
|  | |
| MultipleAlignment | sampleColumns (int colNum, int numConsecutiveCols) |
|  | |
| void | load (String fileName) throws IOException, ParseException |
|  | |
| void | load (RandomAccessFile handle, List< String > sequencesToLoad) throws IOException, ParseException |
|  | |
| void | load (RandomAccessFile handle, int referenceStart, int referenceEnd) throws IOException, ParseException |
|  | |
| void | load (RandomAccessFile handle, int referenceStart, int referenceEnd, List< String > sequencesToLoad) throws IOException, ParseException |
|  | |
| void | load (String fileName, int referenceStart, int referenceEnd) throws IOException, ParseException |
|  | |
| IntervalTree< Long > | getIndex () |
|  | |
| void | writeIndex (String indexFileName) throws IOException |
|  | |
| void | createIndex (String alignmentFile) throws IOException |
|  | |
| void | setBlocks (List< MAFMultipleAlignmentBlock > blocks) |
|  | |
| void | addBlock (MAFMultipleAlignmentBlock block) throws IllegalArgumentException |
|  | |
| void | addBlocks (List< MAFMultipleAlignmentBlock > blocks) throws IllegalArgumentException |
|  | |
| void | addBlocks (Iterator< MAFMultipleAlignmentBlock > blockIt) throws IllegalArgumentException |
|  | |
| void | append (MAFAlignment mafAln) throws IllegalArgumentException |
|  | |
| void | clear () |
|  | |
| Iterator  < MAFMultipleAlignmentBlock > | getBlockIterator () |
|  | |
| void | loadIndex (String idxFile) throws IOException |
|  | |
| Public Member Functions inherited from broad.core.multiplealignment.MultipleAlignment | |
|  | MultipleAlignment () |
|  | |
|  | MultipleAlignment (List<?extends MultipleAlignment > alnBlocks, String ref, String[] seqs) |
|  | |
| void | encode () |
|  | |
| void | encode (boolean distinguishMissingSequence) |
|  | |
| void | encodeAsMatrix () |
|  | |
| void | reverse () |
|  | |
| int | length () |
|  | |
| Map< String, Short > | getColumn (int i) |
|  | |
| Map< String, Matrix > | getColumnsAsVector (int start, int number) throws ArrayIndexOutOfBoundsException |
|  | |
| Map< String, Matrix > | getAsMatrix () |
|  | |
| void | addShortEncodedColumn (Map< String, Short > col) |
|  | |
| void | addShortEncodedColumnMatrix (Map< String, Matrix > col) |
|  | |
| void | addShortEncodedRegion (Map< String, short[]> region) |
|  | |
| char | decodeShort (Short nucleotide) |
|  | |
| void | addSequence (AlignedSequence seq) |
|  | |
| void | addSequences (List< AlignedSequence > sequences) |
|  | |
| boolean | isEmpty () |
|  | |
| void | setIOHelper (MultipleAlignmentIO maio) |
|  | |
| void | write (BufferedWriter bw) throws IOException |
|  | |
| void | write (BufferedWriter bw, List< String > order) throws IOException |
|  | |
| MultipleAlignment | sampleColumns (int colNum, int numConsecutiveCols) |
|  | |
| void | permuteColumns () |
|  | |
| float | getScore () |
|  | |
| void | setScore (float score) |
|  | |
| AlignedSequence | getAlignedSequence (String containingSequenceId) |
|  | |
| List< AlignedSequence > | getAlignedSequences () |
|  | |
| List< String > | getAlignedSequenceIds () |
|  | |
| boolean | overlaps (SequenceRegion region) |
|  | |
| String | getReferenceId () |
|  | |
| void | setReferenceId (String referenceId) |
|  | |
| int | getReferenceStart () |
|  | |
| AlignedSequence | getReference () |
|  | |
| int | getReferenceEnd () |
|  | |
| boolean | isRefGapped () |
|  | |
| void | setRefGapped (boolean isRefGapped) |
|  | |
| MultipleAlignment | getConcatenatedSubAlignment (String refId, List<?extends GenomicAnnotation > annotations) |
|  | |
| void | compress () |
|  | |
| void | remove (List< String > removeList) |
|  | |
| MultipleAlignment | getSubAlignment (int refStart, int refEnd, boolean reverse) |
|  | |
| int | getNumberOfAligningSequences () |
|  | |
| MultipleAlignment | getSubAlignment (String refId, int refStart, int refEnd, boolean reverse) |
|  | |
| void | append (MultipleAlignment other) |
|  | |
| List< int[]> | getUngappedReferenceIslands () |
|  | |
| List< int[]> | getUngappedSequenceReferenceIslands () |
|  | |
| void | introduceGaps (boolean canGapReference, int maxNumberOfGaps) throws IllegalAccessException |
|  | |

|  |  |
| --- | --- |
| Static Public Member Functions | |
| static void | main (String[] args) throws IOException, ParseException |
|  | |

|  |  |
| --- | --- |
| Static Public Attributes | |
| static String | USAGE |
|  | |
| Static Public Attributes inherited from broad.core.multiplealignment.MultipleAlignment | |
| static final short | A\_CODE = Sequence.SHORT\_ENCODED\_A |
|  | |
| static final short | C\_CODE = Sequence.SHORT\_ENCODED\_C |
|  | |
| static final short | G\_CODE = Sequence.SHORT\_ENCODED\_G |
|  | |
| static final short | T\_CODE = Sequence.SHORT\_ENCODED\_T |
|  | |
| static final short | GAP\_CODE = Sequence.SHORT\_ENCODED\_GAP |
|  | |

|  |  |
| --- | --- |
| Protected Member Functions | |
| void | setAlignedSequences (List< String > seqIds) |
|  | |
| Protected Member Functions inherited from broad.core.multiplealignment.MultipleAlignment | |
| MultipleAlignmentIO | getIOHelper () |
|  | |
| Iterator< AlignedSequence > | getAlignedSequences (String refId, int refStart, int refEnd, boolean reverse) |
|  | |

|  |  |
| --- | --- |
| Additional Inherited Members | |
| Static Protected Attributes inherited from broad.core.multiplealignment.MultipleAlignment | |
| static int | UNGAPPED\_ALPHABET\_SIZE = 4 |
|  | |

## Constructor & Destructor Documentation

|  |  |  |  |  |
| --- | --- | --- | --- | --- |
| broad.core.multiplealignment.MAFAlignment.MAFAlignment | ( |  | ) |  |

Here is the caller graph for this function:

|  |  |  |  |  |  |
| --- | --- | --- | --- | --- | --- |
| broad.core.multiplealignment.MAFAlignment.MAFAlignment | ( | String | *indexFile* | ) | throws IOException, ParseException |

Here is the call graph for this function:

## Member Function Documentation

|  |  |  |  |  |  |
| --- | --- | --- | --- | --- | --- |
| void broad.core.multiplealignment.MAFAlignment.addBlock | ( | MAFMultipleAlignmentBlock | *block* | ) | throws IllegalArgumentException |

Here is the caller graph for this function:

|  |  |  |  |  |  |
| --- | --- | --- | --- | --- | --- |
| void broad.core.multiplealignment.MAFAlignment.addBlocks | ( | List< MAFMultipleAlignmentBlock > | *blocks* | ) | throws IllegalArgumentException |

Here is the caller graph for this function:

|  |  |  |  |  |  |
| --- | --- | --- | --- | --- | --- |
| void broad.core.multiplealignment.MAFAlignment.addBlocks | ( | Iterator< MAFMultipleAlignmentBlock > | *blockIt* | ) | throws IllegalArgumentException |

Here is the call graph for this function:

|  |  |  |  |  |  |
| --- | --- | --- | --- | --- | --- |
| void broad.core.multiplealignment.MAFAlignment.addSequence | ( | AlignedSequence | *seq* | ) |  |

|  |  |  |  |  |  |
| --- | --- | --- | --- | --- | --- |
| void broad.core.multiplealignment.MAFAlignment.addSequences | ( | List< AlignedSequence > | *sequences* | ) |  |

|  |  |  |  |  |  |
| --- | --- | --- | --- | --- | --- |
| void broad.core.multiplealignment.MAFAlignment.addShortEncodedColumn | ( | Map< String, Short > | *col* | ) |  |

|  |  |  |  |  |  |
| --- | --- | --- | --- | --- | --- |
| void broad.core.multiplealignment.MAFAlignment.addShortEncodedRegion | ( | Map< String, short[]> | *region* | ) |  |

|  |  |  |  |  |  |
| --- | --- | --- | --- | --- | --- |
| void broad.core.multiplealignment.MAFAlignment.append | ( | MAFAlignment | *mafAln* | ) | throws IllegalArgumentException |

Here is the call graph for this function:

|  |  |  |  |  |
| --- | --- | --- | --- | --- |
| void broad.core.multiplealignment.MAFAlignment.clear | ( |  | ) |  |

|  |  |  |  |  |
| --- | --- | --- | --- | --- |
| void broad.core.multiplealignment.MAFAlignment.compress | ( |  | ) |  |

Here is the caller graph for this function:

|  |  |  |  |  |  |
| --- | --- | --- | --- | --- | --- |
| boolean broad.core.multiplealignment.MAFAlignment.contains | ( | LightweightGenomicAnnotation | *annotation* | ) |  |

Here is the call graph for this function:

|  |  |  |  |  |  |
| --- | --- | --- | --- | --- | --- |
| void broad.core.multiplealignment.MAFAlignment.createIndex | ( | String | *alignmentFile* | ) | throws IOException |

Here is the call graph for this function:

|  |  |  |  |  |
| --- | --- | --- | --- | --- |
| void broad.core.multiplealignment.MAFAlignment.encode | ( |  | ) |  |

|  |  |  |  |  |
| --- | --- | --- | --- | --- |
| void broad.core.multiplealignment.MAFAlignment.encodeAsMatrix | ( |  | ) |  |

|  |  |  |  |
| --- | --- | --- | --- |
| AlignedSequence broad.core.multiplealignment.MAFAlignment.getAlignedSequence | ( | String | *sequenceId*, |
|  |  | boolean | *fillInGapsBetweenBlocks* |
|  | ) |  |  |

Here is the call graph for this function:

Here is the caller graph for this function:

|  |  |  |  |  |  |
| --- | --- | --- | --- | --- | --- |
| AlignedSequence broad.core.multiplealignment.MAFAlignment.getAlignedSequence | ( | String | *sequenceId* | ) |  |

Here is the call graph for this function:

|  |  |  |  |  |
| --- | --- | --- | --- | --- |
| List<String> broad.core.multiplealignment.MAFAlignment.getAlignedSequenceIds | ( |  | ) |  |

Here is the caller graph for this function:

|  |  |  |  |  |  |
| --- | --- | --- | --- | --- | --- |
| List<AlignedSequence> broad.core.multiplealignment.MAFAlignment.getAlignedSequences | ( | boolean | *fillGapsBetweenBlocks* | ) |  |

Here is the call graph for this function:

|  |  |  |  |  |
| --- | --- | --- | --- | --- |
| List<AlignedSequence> broad.core.multiplealignment.MAFAlignment.getAlignedSequences | ( |  | ) |  |

Here is the caller graph for this function:

|  |  |  |  |  |
| --- | --- | --- | --- | --- |
| Iterator<MAFMultipleAlignmentBlock> broad.core.multiplealignment.MAFAlignment.getBlockIterator | ( |  | ) |  |

|  |  |  |  |  |  |
| --- | --- | --- | --- | --- | --- |
| Map<String, Short> broad.core.multiplealignment.MAFAlignment.getColumn | ( | int | *i* | ) |  |

Assumes that encode has been called and each aligned sequence is encoded

Parameters
:   |  |  |
    | --- | --- |
    | int | - alignment column |

Here is the caller graph for this function:

|  |  |  |  |
| --- | --- | --- | --- |
| Map<String, Matrix> broad.core.multiplealignment.MAFAlignment.getColumnsAsVector | ( | int | *start*, |
|  |  | int | *number* |
|  | ) |  |  |

Here is the call graph for this function:

|  |  |  |  |  |
| --- | --- | --- | --- | --- |
| MAFHeader broad.core.multiplealignment.MAFAlignment.getHeader | ( |  | ) |  |

|  |  |  |  |  |
| --- | --- | --- | --- | --- |
| IntervalTree<Long> broad.core.multiplealignment.MAFAlignment.getIndex | ( |  | ) |  |

|  |  |  |  |  |
| --- | --- | --- | --- | --- |
| int broad.core.multiplealignment.MAFAlignment.getReferenceEnd | ( |  | ) |  |

Here is the caller graph for this function:

|  |  |  |  |  |
| --- | --- | --- | --- | --- |
| int broad.core.multiplealignment.MAFAlignment.getReferenceStart | ( |  | ) |  |

Here is the caller graph for this function:

|  |  |  |  |
| --- | --- | --- | --- |
| MAFAlignment broad.core.multiplealignment.MAFAlignment.getSubAlignment | ( | int | *refStart*, |
|  |  | int | *refEnd*, |
|  |  | boolean | *reverse* |
|  | ) |  |  |

Here is the call graph for this function:

|  |  |  |  |  |
| --- | --- | --- | --- | --- |
| boolean broad.core.multiplealignment.MAFAlignment.isEmpty | ( |  | ) |  |

Here is the caller graph for this function:

|  |  |  |  |  |
| --- | --- | --- | --- | --- |
| int broad.core.multiplealignment.MAFAlignment.length | ( |  | ) |  |

Here is the caller graph for this function:

|  |  |  |  |  |  |
| --- | --- | --- | --- | --- | --- |
| void broad.core.multiplealignment.MAFAlignment.load | ( | String | *fileName* | ) | throws IOException, ParseException |

Here is the caller graph for this function:

|  |  |  |  |
| --- | --- | --- | --- |
| void broad.core.multiplealignment.MAFAlignment.load | ( | RandomAccessFile | *handle*, |
|  |  | List< String > | *sequencesToLoad* |
|  | ) |  | throws IOException, ParseException |

Here is the call graph for this function:

|  |  |  |  |
| --- | --- | --- | --- |
| void broad.core.multiplealignment.MAFAlignment.load | ( | RandomAccessFile | *handle*, |
|  |  | int | *referenceStart*, |
|  |  | int | *referenceEnd* |
|  | ) |  | throws IOException, ParseException |

Here is the call graph for this function:

|  |  |  |  |
| --- | --- | --- | --- |
| void broad.core.multiplealignment.MAFAlignment.load | ( | RandomAccessFile | *handle*, |
|  |  | int | *referenceStart*, |
|  |  | int | *referenceEnd*, |
|  |  | List< String > | *sequencesToLoad* |
|  | ) |  | throws IOException, ParseException |

Here is the call graph for this function:

|  |  |  |  |
| --- | --- | --- | --- |
| void broad.core.multiplealignment.MAFAlignment.load | ( | String | *fileName*, |
|  |  | int | *referenceStart*, |
|  |  | int | *referenceEnd* |
|  | ) |  | throws IOException, ParseException |

Here is the call graph for this function:

|  |  |  |  |  |  |
| --- | --- | --- | --- | --- | --- |
| void broad.core.multiplealignment.MAFAlignment.loadIndex | ( | String | *idxFile* | ) | throws IOException |

Here is the caller graph for this function:

|  |  |  |  |  |  |
| --- | --- | --- | --- | --- | --- |
| static void broad.core.multiplealignment.MAFAlignment.main | ( | String[] | *args* | ) | throws IOException, ParseException |

static

Here is the call graph for this function:

|  |  |  |  |  |  |
| --- | --- | --- | --- | --- | --- |
| boolean broad.core.multiplealignment.MAFAlignment.overlaps | ( | LightweightGenomicAnnotation | *annotation* | ) |  |

Here is the call graph for this function:

|  |  |  |  |  |
| --- | --- | --- | --- | --- |
| void broad.core.multiplealignment.MAFAlignment.reverse | ( |  | ) |  |

|  |  |  |  |
| --- | --- | --- | --- |
| MultipleAlignment broad.core.multiplealignment.MAFAlignment.sampleColumns | ( | int | *colNum*, |
|  |  | int | *numConsecutiveCols* |
|  | ) |  |  |

Here is the call graph for this function:

|  |  |  |  |  |  |
| --- | --- | --- | --- | --- | --- |
| void broad.core.multiplealignment.MAFAlignment.setAlignedSequences | ( | List< String > | *seqIds* | ) |  |

protected

|  |  |  |  |  |  |
| --- | --- | --- | --- | --- | --- |
| void broad.core.multiplealignment.MAFAlignment.setBlocks | ( | List< MAFMultipleAlignmentBlock > | *blocks* | ) |  |

Here is the call graph for this function:

|  |  |  |  |  |  |
| --- | --- | --- | --- | --- | --- |
| MultipleAlignment broad.core.multiplealignment.MAFAlignment.toMultipleAlignment | ( | boolean | *fillGapsBetweenBlocks* | ) |  |

Here is the call graph for this function:

|  |  |  |  |  |
| --- | --- | --- | --- | --- |
| MultipleAlignment broad.core.multiplealignment.MAFAlignment.toMultipleAlignment | ( |  | ) |  |

|  |  |  |  |  |  |
| --- | --- | --- | --- | --- | --- |
| void broad.core.multiplealignment.MAFAlignment.write | ( | BufferedWriter | *bw* | ) | throws IOException |

Here is the call graph for this function:

|  |  |  |  |  |  |
| --- | --- | --- | --- | --- | --- |
| void broad.core.multiplealignment.MAFAlignment.writeIndex | ( | String | *indexFileName* | ) | throws IOException |

## Member Data Documentation

String broad.core.multiplealignment.MAFAlignment.USAGE

static

**Initial value:**

= "Usage: MAFAlignment TASK=<task\_num> <task\_args>\n" +

"\tTasks:" +

"\n\t\t1. Extract alignments -in <input file> -out <output file, default writes to standard out> -start <chromosome start> -end <chromosome end> [-compress <if this flag is present, the alignment output will contain no reference alignment gaps> -outformat <Specify FASTA, PHYLIP or PHYLIPSEQ if a format other than MAF is decired for the output> -noPadding (include this flag if no padding betweein non contiguous MAF blocks is desired, not that any reference to alignment positions will be lost) ]\n" +

"n\t\t2. Extract regions -in <alignment file in MAF format> -out <output file> -annotations <Anntation file (BED format by default)> [-format <[BED], SIMPLE,GFF>] -seqsToLoad <List of sequences to load or all if non specified> -compress <if the result should be devoid of reference gaps> -outformat <FASTA, PHYLIP, SEQPHYLIP, if none is specifed the default is MAF>]\n" +

"n\t\t3. Create index file -in <alignment file in MAF format> -out <Index file name or standard output>\n"+

"n\t\tExtractBed. Extract segements from a bed from different chrs : similar to extract region but will extract seq of each BED line and will generate an outfile for each exon specified in the BED -in <Directory of chr alignment file in MAF format ; dir/chrX.maf> -out <output prefix (will generate multiple out files, one for each record in the BED)> -annotations <Anntation file (BED format by default)> [-format <[BED], SIMPLE,GFF>] -seqsToLoad <List of sequences to load or all if non specified> -compress <if the result should be devoid of reference gaps> -outformat <FASTA, PHYLIP, SEQPHYLIP, if none is specifed the default is MAF>] -fullBed <flag if you want each exon to be reported>\n"

---

The documentation for this class was generated from the following file:

- java/broad/core/multiplealignment/MAFAlignment.java


---

Generated on Fri Aug 15 2014 15:10:54 for ESAT by  

 1.8.7
